# Priming defense by transiently suppressing plant susceptibility genes in ornamental crops: A novel strategy for post-harvest diseases management

**DOI:** 10.3389/fpls.2022.1025165

**Published:** 2022-09-15

**Authors:** Xintong Liu, Yuling Bai, Zhao Zhang

**Affiliations:** ^1^ Beijing Key Laboratory of Development and Quality Control of Ornamental Crops, Department of Ornamental Horticulture, China Agricultural University, Beijing, China; ^2^ Plant Breeding, Wageningen University & Research, Wageningen, Netherlands

**Keywords:** ornamental crops, disease resistance, susceptibility genes, *S*-genes, postharvest diseases

## Introduction: Manipulating host susceptibility genes for resistance

The susceptibility genes (*S*-genes) refer to the plant genes required by phytopathogens to facilitate their compatible interaction with hosts (Pavan et al., 2010; Koseoglou et al., 2021). The pathogens exploit host *S*-genes for their invasion, consequently, the loss of function of *S*-genes disturb the compatible interaction and therefore lead to resistance. The resistance mediated by mutant alleles of *S*-genes have been reported in various plant species, and has been successfully applied to breeding for many years.

The *S*-genes, based on their involvement in different stages of infection, were classified into several functional groups (van Schie and Takken, 2014; Koseoglou et al., 2021). The first group includes genes required for early pathogen infestation or to promote pathogen infestation. MLO is one of the best-known typical members of the first group, of which a recessive mutant with powdery mildew (PM) resistance in barley was developed decades ago and this mutant is still used in European spring barley and confers resistance to all PM races in the field (Freisleben and Lein, 1942; Jørgensen, 1992; Dreiseitl, 2020). The mutation of *MLO* with CRISPR-Cas9 mediated system have been applied to confer PM resistance in several plant species including wheat (Shan et al., 2013), tomato (Nekrasov et al., 2017) and grape (Malnoy et al., 2016). The second group of *S*-genes encodes negative regulators in plant immune signaling. These genes are involved in regulating the levels of the phytohormones, and their crosstalk, such as salicylic acid (SA) and jasmonic acid (JA). For example, the recessive mutation of *CESA3*, one of the cellulose synthesis-related genes, showed an increased level of JA, abscisic acid (ABA) and ethylene (ET), resulting in enhanced resistance to several pathogens (Ellis et al., 2002; Kumar and Turner, 2015). CESA4, CESA7 and CESA8 are also associated with susceptibility to pathogens and their corresponding mutants show increased resistance to both pathogenic fungus and bacteria (Hernández-Blanco et al., 2007). The third group of *S*-genes is associated with the nutrition of pathogenic bacteria, such as metabolite biosynthesis and sugar transport, endogenous replication and cell expansion. In rice, the loss of function of *SWEET14* increased the resistance to bacterial blight (Li et al., 2012). Recently, the plant transporter genes that targeted by bacterial pathogens for their effector translocation were suggested as a new functional group of *S*-genes (Koseoglou et al., 2021).

## Translating knowledge from the laboratory to the field: Current difficulties in using *S*-genes for disease control of ornamental crops

The business of ornamental horticulture makes tens of billions of dollars annually, and pathogenic microorganisms pose a huge hazard to this industry worldwide. The rose production, for example, requires over $5,000 worth of pesticide spraying per hectare per year to control the disease. Therefore, the manipulation of *S*-genes for disease control of ornamental crops would have great potential to create significant value.

**Figure 1:**
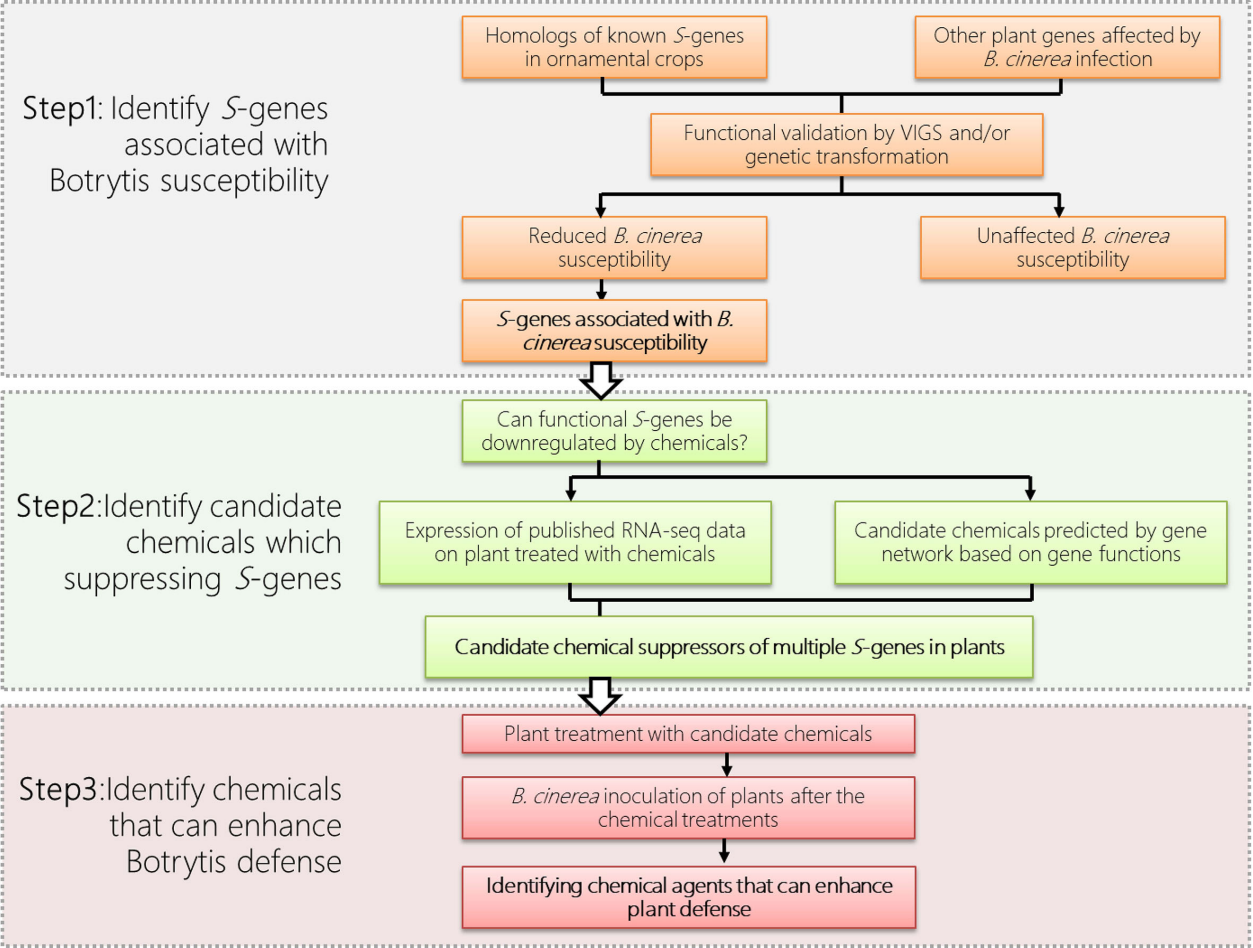
Priming defense by chemically suppressing plant *S*-genes in ornamental crops, using gray mold disease (caused by *Botrytis cinerea*) as an example.

To utilize *S*-gene for defense in ornamental plants, the first step is the identification of those functional *S*-genes from the ornamental plant (**Figure 1**). As *S*-genes are often universal among different plant species, we can therefore use information from model plants or other crops and identify homologs of *S*-genes from the available genomic data of the ornamental crops of interest. In addition, *S*-genes, especially those encoding defense suppressors are usually induced by pathogens during the infection process. Thus plant transcriptome data under infection of various pathogens are also important clues for our identification of functional *S*-genes.

Although the homologous genes are often thought to be functionally conserved across species, not all homologs in a certain *S*-gene family will function as susceptibility factors in all plant species. The involvement of each homolog of an *S*-gene family demands a case-by-case assessment of their usefulness for resistance in each plant species. Therefore, the identification of functional *S*-gene homologs in each species is a challenge. In addition to RNAi strategy using stable transformation, a virus-induced gene silencing (VIGS) has also been demonstrated to be a rapid and effective method for transient knockdown of gene expression in various other plant species, including many ornamental plants, such as petunia (Spitzer et al., 2007), rose (Yan et al., 2018; Cao et al., 2019), gladiolus (Zhong et al., 2014), gerbera (Deng et al., 2012), and lily (Wu et al., 2018). With VIGS, we can quickly compare the differences in pathogen susceptibility, after partial loss of function of the *S*-gene in ornamentals. For instance, the report has identified 19 MLO genes in rose. Of these, VIGS of *RhMLO1* and *RhMLO2*, are required for infection by *Podosphaera pannosa* and suggest their potential as susceptibility targets for powdery mildew resistance breeding (Fang et al., 2021).

To manipulate the interesting *S*-genes for disease resistance in ornamentals, methods are needed to artificially downregulate/disable these genes. However, there are three major obstacles to achieving this goal: First, although loss-of-function mutations of *S*-genes could be generated *via* various approaches, such as using natural alleles from genetic resources, stable RNAi, and newly emerging gene editing techniques, these approaches are technically challenging in many other ornamental crops due to their polyploid genomes and the lack of efficient transformation techniques. Second, it is challenging to downregulate multiple *S*-genes simultaneously in these plants to improve the resistance effect. Third, there are severe limits to developing and propagating genetically modified crops in many countries, especially in Europe. All of these issues represent barriers to the successful use of *S*-gene in ornamental crops (Smulders et al., 2019; Gahlaut et al., 2021).

## Suppressing (multiple) plant *S*-genes by using ‘priming’ agents: A novel strategy

Defense priming represents a “warm-up” strategy for plant resistance. During this process, the host plant enters a priming phase triggered by stimuli that act as an alarm from pathogens or beneficial microbes. In addition, the priming agents in the absence of pathogens, such as jasmonic acid (JA), salicylic acid (SA), *β*-aminobutyric acid (BABA), benzothiadiazole (BTH), hexanoic acid (Hx), chitosan, tricarboxylates, ergosterol and many other natural or synthetic agents can promote plant defense (Laquitaine et al., 2006; van Schie and Takken, 2014; Mauch-Mani et al., 2017; Balmer et al., 2018; Duanis-Assaf et al., 2022). Once the host plant enters the priming phase, only mild changes occur at the transcriptional and/or epigenetic level without the dramatic induction of many defense genes. Therefore, the priming stage is a low-cost strategy for the host plant. This stage can last for more than 10 days (almost spanning the entire transportation and shelf period of cut flowers) or throughout the plant lifecycle (Mauch-Mani et al., 2017).

Upon subsequent pathogen infection, the primed plant mounts a faster and/or stronger defense response than a non-primed plant, resulting in significantly increased resistance. Most studies of this process have focused on the increased expression of resistance (*R*) genes upon pre-exposure to a priming stimulus and subsequent pathogen infection; however, numerous genes are also substantially downregulated, this includes many known *S*-genes of plants (Verhagen et al., 2004; Finiti et al., 2014). Therefore, it is clear that priming agents could potentially be used to downregulate *S*-genes for enhanced resistance *via* the defense priming strategy.

Evidence has shown that *S*-genes can be silenced when plants are treated with chemicals. For example, in strawberry (Landi et al., 2017), 8 homologs of known *S*-genes (including *PLP*s,*WRKY57*,*PG1*,*MPK4*,*SWEET14*,*Cel1/2*) are differentially expressed upon treatment of BTH and chitosan at 6, 12, or 24 hours post treatment (hpt). In particular, four of them are significantly downregulated in BTH and seven of them are significantly downregulated in chitosan, suggesting that BTH and chitosan are candidate agents which can enhance *B. cinerea* resistance by suppressing the expression of multiple *S*-genes. Assumingly, agents identified by gene expression profiling that inhibit the expression of *S*-gene can subsequently be applied to plants to induce plant defense. In fact, the postharvest application of BTH effectively reduced latent infections and induced resistance to diseases in fruit and vegetables, such as strawberries (Feliziani et al., 2015), peaches (Liu et al., 2005) and melons (Feliziani et al., 2015).

The above examples illustrate that conventional priming agents (e.g. BTH) can function as suppressors to down-regulate expression of certain *S*-genes leading to resistance in plants. To apply this concept on priming defense to postharvest diseases in ornamental crops, we propose to transiently suppress the expression of certain plant *S*-genes, by using various suppressor agents (**Figure 1**). In addition to BTH, the analysis of the continuously generated ‘Omics’ data allows us to high-throughput screen for novel suppressor agents for the regulation of *S*-gene and modulation of plant resistance. These ‘Omics’ data include publicly available RNA-seq dataset of plants upon the treatment of chemicals, including Hx (Finiti et al., 2014), polyamines (Seifi et al., 2019), BTH (Cheng et al., 2018), chitosan (Landi et al., 2017), BABA (Bengtsson et al., 2014), phosphite (Burra et al., 2014) and various phytohormones (Ran et al., 2018).

As in the examples given in **Figure 1**, the functional *S*-genes and their suppressor agents can be identified through the bioinformatic, reverse genetic and phytopathological approaches. The identified functional *S*-genes (**Figure 1** Step1) can be used as ‘markers’ to screen the candidate suppressor agents based on their expression (**Figure 1** Step 2). The function of candidate suppressor agents can subsequently be verified by their application to plants and pathogen assays (**Figure 1** Step 3). Multiple agents can also be used in combination to suppress the expression of multiple *S*-genes to enhance disease resistance in ornamentals while taking into account the cost of application and environmental sustainability issues. Last but not the least, the molecular mechanisms of the newly identified suppressor agents should be further investigated.

## Prospects

Recent research suggested various natural or synthetic agents could be used to downregulate multiple S-genes simultaneously. These findings prompted us to explore the feasibility of achieving induced defense priming in ornamental crops by downregulating their S-genes transiently by application of proper priming agents, which can be applied to pot-plants and cut-flowers during the period of transportation and on-shelf stage. Here we propose a strategy for controlling postharvest diseases in ornamental crops by transiently suppressing (multiple) plant *S*-genes to prime defense (**Figure 1**). To this end, based on the multi-omic dataset from a publicly available database or generated by experiments, the potential S-genes will be identified and evaluated for their expression pattern upon the treatment of certain agents, such as natural compounds or natural plant hormones. Agents that repress the expression of (multiple) S-genes will be applied to the plant to test their role in promoting plant defense. It is worth noting that S-genes identified from one plant species can be applied to another, with potentially different diseases. This approach means providing a strategy for broad-spectrum disease resistance in various ornamental plants, which will save the economic value of these crops from disease devastation. Another attractive feature of eliciting priming for plant protection is that it avoids the use of environmentally problematic fungicides; the use of these products can also lead to the appearance of fungal strains resistant to these fungicides. We expect that the knowledge about S-genes and defense priming obtained in our analysis will increasingly be put into practice in the field, thereby improving sustainable agriculture.

## Author contributions

YB, ZZ, and XL conceived and wrote the paper. All authors contributed to the article and approved the submitted version.

## Funding

This study was supported by the National Natural Science Foundation of China (grant number 31972444) to ZZ. The project is further supported by the Construction of Beijing Science and Technology Innovation and Service Capacity in Top Subjects (CEFF-PXM2019_014207_000032).

## Conflict of interest

The authors declare that the research was conducted in the absence of any commercial or financial relationships that could be construed as a potential conflict of interest.

## Publisher’s note

All claims expressed in this article are solely those of the authors and do not necessarily represent those of their affiliated organizations, or those of the publisher, the editors and the reviewers. Any product that may be evaluated in this article, or claim that may be made by its manufacturer, is not guaranteed or endorsed by the publisher.
